# Effects of Metoprolol and Nebivolol on Exercise Blood Pressure in Patients with Mild Hypertension

**DOI:** 10.1155/2013/608683

**Published:** 2013-10-29

**Authors:** Huseyin Ugur Yazici, Hande Ozduman, Yuksel Aydar, Alparslan Birdane

**Affiliations:** ^1^Department of Cardiology, School of Medicine, Eskişehir Osmangazi University, 26000 Eskişehir, Turkey; ^2^Department of Anatomy, School of Medicine, Eskişehir Osmangazi University, Eskişehir, Turkey

## Abstract

*Objectives*. We planned to compare the impact of two beta blockers, metoprolol and nebivolol, on arterial blood pressure during exercise in patients with mild hypertension. *Methods*. A total of 60 patients (13 males, 47 females; mean age: 54.3 ± 10.7 years) were enrolled in the present study. The patients were randomly selected to receive either nebivolol 5 mg/day (*n* = 30) or metoprolol 50 mg/day (*n* = 30) for 8 weeks. At the end of the 8th week, each of the patients received exercise stress test according to Bruce protocol and their blood pressures were remeasured after rest, exercise, and recovery. *Results*. Blood pressures were determined to be similar between metoprolol and nebivolol groups during rest, exercise, and recovery periods. Metoprolol and nebivolol achieved similar reductions in blood pressures during rest and exercise. However, five patients in nebivolol group and four patients in metoprolol group developed exaggerated BP response to exercise but the difference between metoprolol and nebivolol was not meaningful (*P* = 0.37). *Conclusion*. The results of the present study showed that metoprolol and nebivolol established comparable effects on the control of blood pressures during exercise in the patients with mild hypertensions.

## 1. Introduction

Development of exaggerated BP response to exercise is shown to be associated with increased cardiovascular morbidity and mortality [1–3]. Sudden rise in blood pressure (BP) in patients with hypertension due to mental and physical stresses during daily activities is reported to be responsible for the left ventricular hypertrophy and increased risk for developing acute cardiovascular event [4–6]. Therefore, the antihypertensive drugs that would be used in the treatment of hypertension are required to be able to control BP both during rest and exercise. Moreover, beta blockers are demonstrated to manage BP better during both rest and exercise than other antihypertensive drugs [7–9]. However, whether beta blockers possess discrete effect among themselves has not been well studied.

Beta blockers have been used in the treatment of hypertension for years and they are still the first line of drugs suggested [10, 11]. Metoprolol and nebivolol are both beta-1-blockers and these drugs are considered prominent members of this group. Nebivolol, a third generation of beta blockers, improves endothelial functions and exerts its vasodilatory effect by enhancing NO release [12–16]. Nebivolol is expected to control BP more effectively since it increases peripheral vasodilatation.

We aimed to evaluate the effects of nebivolol and metoprolol on arterial BP during exercise.

## 2. Methods

### 2.1. Patients

We randomly included 30 patients for metoprolol group and 30 patients for nebivolol group. A total of 60 patients (13 males, 47 females; mean age: 54.3 ± 10.7 years) were enrolled in the present study. The study was approved by the local ethics committee; the patients were informed and written consents from them were also obtained. All procedures were conducted in accordance with the Declaration of Helsinki (1964).

### 2.2. Inclusion Criteria

The patients diagnosed newly with low-risk hypertensions at stage 1 were included in this study. The stage of hypertension was determined according to Guidelines for the Management of Arterial Hypertension approved by the European Society of Hypertension and by the European Society of Cardiology in 2007 [11]. Stage 1 hypertension was defined as the presence of systolic BP of >140 mmHg but <160 mmHg or the occurrence of diastolic BP of >90 mmHg but <100 mmHg obtained from more than two blood measurements taken at different times for the same patient.

### 2.3. Exclusion Criteria

The following patients were excluded from the current study: patients possessing middle or high-risk arterial hypertension, having hypertension beyond stage 1, showing fasting blood glucose of >126 mg/dL, previously taking any kind of antihypertensive medication, suffering from cardiovascular diseases, diabetes mellitus, chronic obstructive pulmonary disease, significant systemic disease, and atrial fibrillation, showing evidence of myocardial ischemia at exercise stress test, having concomitant diseases that might adversely impair his/her exercise capacity (musculoskeletal diseases), or showing contraindications to b-blocker therapy (e.g., advanced heart block).

### 2.4. Study Protocol

The patients were randomized at a 1 : 1 ratio to receive metoprolol succinate (50 mg daily) (Beloc Zok, AstraZeneca, UK) or nebivolol (5 mg daily) (Vasoxen, Ibrahim Ethem Ulagay-Menarini, Italy) once a day in the morning for 8 weeks. The patients were carefully checked for the control of their BP and heart rate and for their compliance with medication at 2, 4, and 8 weeks of the treatment. The patients were asked not to use any kind of other drugs with known impact on the cardiovascular system throughout the study. Moreover, blood pressure was measured in the sitting position after 5 min of rest. The median value of two consecutive measurements taken at 2 min intervals on two different days was used for the study. The measurements of the BP were completed on the right arm of the patients using a sphygmomanometer with an appropriate cuff size (Sentron, CR Bard Inc., Lombard, IL, USA). Successful antihypertensive therapy was defined as the establishment of blood pressure <140/90 (systolic/diastolic) at the end of the eight-week treatment.

### 2.5. Exercise Stress Test

At the end of the 8th week, the patients received EST according to the standard Bruce protocol (QuintonTreadmill system, Quinton, Inc., Bothell, WA, USA) [17]. The patients were encouraged to continue exercising till experiencing limiting symptoms. All the patients completed the test successfully and no test-associated complications were developed. While resting BP was taken in sitting position just before exercise, exercise BP was taken every two minutes in standing position during exercise. Recovery BP was completed at the 3rd and 5th minutes after the end of the exercise in, again, sitting position. Men with a peak exercise systolic BP of 210 or more and women with a peak exercise systolic BP of 190 or more were considered to have an “exaggerated” BP response [18].

### 2.6. Statistical Analysis

While continuous variables were expressed as mean ± SD, categorical variables were defined as percentages. While Student's *t*-test was used to compare continuous variables showing normal distribution, Mann-Whitney *U* test was used to compare continuous variables not showing normal distribution. Categorical variables were compared using the chi-square test. A two-tailed paired *t*-test was used to compare continuous variables before and after drug therapy within the same group. For all the tests, a value of *P* < 0.05 was considered to be statistically significant. Statistical analyses were performed using the SPSS 16.0 Statistical Package Program for Windows (SPSS Inc., Chicago, IL, USA).

## 3. Results 

Overall, 66 patients were enrolled in this study at the beginning; however, six patients were left out since two of them stopped taking metoprolol due to undesired side effects and two in metoprolol and two in nebivolol groups did not come to planned visits for their treatments. Consequently, 60 patients (47 females, 13 males) with mean age of 54.3 ± 10.7 were included in the final analyses. On the whole, no significant differences were recorded between the metoprolol and nebivolol groups for their clinical and laboratory features. Main clinical and laboratory features of the patients are illustrated in Table 1.

With regard to prior to therapy, heart rates and systolic and diastolic blood pressures were significantly reduced (*P* < 0.001) in metoprolol and nebivolol groups at the end of the eight-week treatment. The effects of metoprolol and nebivolol on the heart rates and systolic and diastolic blood pressures were comparable. The values of blood pressures and heart rates prior to and after the treatments with metoprolol and nebivolol are summarized in Table 2.

Moreover, exercise BP response was between desired levels at all stages of the exercises in both groups. Systolic and diastolic blood pressures and heart rates at rest, exercise stress test (stages 1, 2, and 3 and peak exercise), and at 3rd and 5th minutes of recovery period were alike between metoprolol and nebivolol groups. The heart rates and systolic and diastolic blood pressures measured during rest, exercise stress test, and recovery periods are recapitulated in Table 3.

We also compared metoprolol and nebivolol groups developing exaggerated BP rates in response to exercise. While successful antihypertensive treatments were achieved in 61.6% of the study population, antihypertensive treatment failed in 38.4% of the patients at the end of the eight-week treatment. In addition, while three of the patients (8.1%) with controlled hypertension developed exaggerated blood pressure, six of the patients (26.1%) with uncontrolled hypertension developed exaggerated blood pressure (*P* = 0.65). The frequency of developing exaggerated blood pressure response in the two groups was similar. Five patients in the nebivolol group and four patients in the metoprolol group developed exaggerated BP response to exercise, but the difference between metoprolol and nebivolol was not meaningful (*P* = 0.37). The features concerning the patients developing normal and exaggerated BP response to exercise are summarized in Table 4.

## 4. Discussion

The results of the present study indicated that both metoprolol and nebivolol possessed similar effect on the BP during exercise and rest in the patients with mild hypertensions. Beta blockers were able to control BP well, and with the use of them, few of the patients (9/60) developed exaggerated BP response to exercise.

The balance between physiologically increased cardiac output and dilatation in peripheral vascular elements in muscles determines BP during exercise. If an increase in cardiac output during exercise is not balanced with peripheral vasodilatation, it causes a sharp rise in systolic BP [5]. Awareness of the exaggerated hypertensive response during exercise is shown to be more valuable than that of BP measured during rest since exaggerated hypertension carries the risk of impairing targeted organs [19, 20]. However, some studies report similar exercise and ambulatory BP [21]. Therefore, exercise stress test, consisting of rest, exercise, and recovery periods, is used to standardize exercise-related occurrence of exaggerated hypertensions [22, 23]. Accordingly, we used treadmill exercise stress test to study the effect of metoprolol and nebivolol on exaggerated hypertensive response during exercise.

Beta blockers are shown to control exercise BP better with respect to other antihypertensive drugs, and few patients are shown to develop exercise-associated exaggerated hypertension against the use of these blockers [7]. Nevertheless, there are limited numbers of studies available on the fact that whether different beta blockers show distinct effect on the control of exercise-associated exaggerated hypertension. In their study, Nodari et al. treated their patients with atenoeol and nebivolol for six months and compared the impact of them on peak exercise BP. Their study showed that both drugs similarly decreased systolic and diastolic BP [24]. Likewise, in another study, Stoschitzky et al. reported that nebivolol, bisoprolol, and carvedilol equally lowered the systolic BP during rest and exercise [25]. Recent studies using metoprolol and nebivolol reported that both drugs were similarly effective in reducing systolic and diastolic BP during rest [26, 27]. An interesting recent study carried out by Kampus et al. documented that the use of metoprolol or nebivolol for one year was comparablly effective in controlling peripheral BP; by contrast, nebivolol was more effective than metoprolol in reducing central aortic and diastolic BP [28]. However, none of these studies investigated and compared the effect of metoprolol and nebivolol on exercise-regulated BP. To our knowledge, this is the first study comparing the effect of metoprolol and nebivolol on exercise-associated increase in BP.

Metoprolol succinate (50 mg/day) and nebivolol (5 mg/day) similarly controlled BP response during rest and exercise in the present study. At the end of the eighth week, the treatment of the patients with these drugs achieved targeted BP response during rest and exercise in most of the patients. Our observations were consistent with other studies investigating the effects of metoprolol and nebivolol [26–28]. Apart from metoprolol and nebivolol, a third generation of beta blockers improves endothelial function through increasing NO discharge [12–16]. Vasodilatory effect of nebivolol is also shown to contribute to its antihypertensive effect [29]. Peripheral vasodilatation, particularly during exercise, is decisively significant for controlling BP. Nebivolol is expected to control BP more efficiently owing to its effect. Systolic and diastolic blood pressures were similar during all stages of exercise stress test in the present study.

We think that the following elements could regulate comparable antihypertensive effects of metoprolol and nebivolol. Exercise increases adrenergic activity. Antiadrenergic activity of metoprolol is reported to be more potent than nebivolol [29]. On the other hand, since nebivolol possesses vasodilatory effect in contrast to metoprolol, this feature may enhance its antihypertensive impact during exercise. Eventually, two distinct features of metoprolol and nebivolol could equilibrate the antihypertensive effect of these drugs, thereby exerting similar control on exercise BP. Another reason for obtaining similar effect with metoprolol and nebivolol on exercise BP could be the present patient population that possessed low-risk hypertension at stage 1. Less endothelial dysfunction is reported to develop in the patients with low-risk hypertension at stage 1 with respect to the patients with moderate risk and severe hypertension [10, 11]. It is possible that since endothelial dysfunction is modest in the present patients, recuperative effect of nebivolol for improving endothelial functions may not be as prominent as needed for reflecting homodynamic parameters that we measured.

Moreover, the metabolic demand for many leisure and routine daily activities in middle-aged individuals is shown to be approximately 5 to 7 metabolic equivalents (METs) [29]. Using Bruce protocol, 4.6, 6.8, and 9.4 METs are consumed at the end of stages 1, 2, and 3, respectively. In the current study, we assessed the BP in response to moderate physical activity in the patients with mild hypertension, and 15% of the patients showed exaggerated BP response to exercise. These observations are consistent with previous studies [7]. Even though it was not statistically significant, the number of the patients developing exaggerated BP response to exercise among the patients treated successfully using antihypertensive treatment was fewer. Our observations suggest that particularly effective control of resting BP should be achieved to prevent development of exaggerated BP response to exercise. The incidence for developing exaggerated BP response to exercise was higher among females in the present study, an observation that might be a result of the classification system that we used here [7, 18].

In conclusion, the results of the present study demonstrated that metoprolol and nebivolol achieved comparable effects on the control of blood pressures during exercise in the patients with mild arterial hypertensions. Current observations suggest that metoprolol and nebivolol can reduce cardiovascular risk via reducing exaggerated BP response to exercise in hypertensive patients.

## Figures and Tables

**Table 1:** Main clinical and laboratory features of the present study population.

	Total (*n* = 60)	Metoprolol (*n* = 30)	Nebivolol (*n* = 30)	*P* value
Age	54.3 ± 10.7	55.7 ± 11.7	52.8 ± 9.5	0.3
Female (*n*, %)	13 (21.6)	7 (23.3)	6 (20)	0.51
Smoking (*n*, %)	17 (28.3)	8 (26.6)	9 (30)	0.5
Family history (*n*, %)	10 (16.6)	5 (16.6)	5 (16.6)	0.6
Hyperlipidemia (*n*, %)	14 (23.3)	6 (20)	8 (26.6)	0.38
Body mass index (kg/m^2^)	27.5 ± 3.4	27.6 ± 3.2	27.2 ± 3.7	0.62
Creatinine (mg/dL)	0.77 ± 0.14	0.79 ± 0.13	0.74 ± 0.15	0.2
Glucose (mg/dL)	94.7 ± 13.6	95.5 ± 14.7	93.9 ± 12.8	0.66
Hemoglobin (gm/dL)	13.6 ± 1.3	13.7 ± 1.3	13.6 ± 1.3	0.92
Left ventricle ejection fraction (%)	69 ± 4.2	68.8 ± 3.8	69.1 ± 4.7	0.79
*E*/*A* ratio	0.93 ± 0.32	0.92 ± 0.28	0.93 ± 0.34	0.91
Left ventricle mass index	107.7 ± 18.1	108.1 ± 12.2	107.2 ± 22.8	0.86

**Table 2:** The values of blood pressures and heart rates prior to and after the treatment with metoprolol and nebivolol in the study population.

	Total (*n* = 60)	Metoprolol (*n* = 30)	Nebivolol (*n* = 30)	*P* value
Systolic blood pressure (mm/Hg)				
Before	152.1 ± 4.9	151.3 ± 3.6	152.8 ± 5.9	0.27**
After	136.4 ± 10.9	134.9 ± 10.5	137.8 ± 11.3	0.3**
*P* value	<0.001*	<0.001*	<0.001*	
Diastolic blood pressure (mm/Hg)				
Before	91.7 ± 3.9	91.1 ± 3.7	92.3 ± 4.1	0.25**
After	82.6 ± 7.1	82.4 ± 6.7	82.7 ± 7.3	0.87**
*P* value	<0.001*	<0.001*	<0.001*	
Heart rate (pulse/min)				
Before	75.7 ± 5.7	75.2 ± 5.7	76.2 ± 5.7	0.5**
After	70.2 ± 5.4	70.3 ± 5.8	70.1 ± 5.1	0.87**
*P* value	<0.001*	<0.001*	<0.001*	
Achieved targeted blood pressure (*n*, %)	37 (61.6)	19 (63.3)	18 (60)	0.5***

*Paired *t*-test; **independent samples *t*-test; ***chi-square test.

**Table 3:** The values of blood pressures and heart rates measured during rest, exercise, and recovery periods in the study population.

	Total (*n* = 60)	Metoprolol (*n* = 30)	Nebivolol (*n* = 30)	*P* value
Systolic blood pressure (mm/Hg)				
Rest	137.4 ± 13.7	135.3 ± 13.5	139.6 ± 13.8	0.22
Stage 1	152 ± 22.5	151.7 ± 20.9	152.3 ± 24.4	0.91
Stage 2	162.7 ± 27.1	160.8 ± 27.1	164.6 ± 27.5	0.61
Stage 3	175.3 ± 29.3	174.3 ± 29.7	176.2 ± 29.7	0.84
Recovery (3 min)	164.4 ± 27.4	167.1 ± 29.8	161.7 ± 25	0.46
Recovery (5 min)	156.5 ± 27.9	158.5 ± 27	154.5 ± 29	0.58
Diastolic blood pressure (mm/Hg)				
Rest	82.6 ± 9.9	83.7 ± 10.6	81.5 ± 9.3	0.39
Stage 1	76 ± 21.2	78.6 ± 21.5	73.3 ± 20.9	0.34
Stage 2	72.9 ± 18.7	76.7 ± 16.5	68.9 ± 20.2	0.12
Stage 3	71.5 ± 19.5	77.3 ± 11.5	66.7 ± 23.4	0.07
Recovery (3 min)	77.9 ± 17	81.8 ± 16.6	74.1 ± 16.7	0.08
Recovery (5 min)	81.9 ± 12.9	83.2 ± 14.1	80.7 ± 11.8	0.45
Heart rate per minute				
Rest	71.7 ± 5.5	72.1 ± 5.3	71.2 ± 5.9	0.63
Stage 1	106.7 ± 16.4	105.2 ± 17.2	108.1 ± 15.7	0.49
Stage 2	122.8 ± 17.2	119.6 ± 16.6	126.3 ± 17.6	0.15
Stage 3	139.5 ± 18.5	140.3 ± 18.1	138.9 ± 19.2	0.82
Recovery (3 min)	118.7 ± 16.3	117.2 ± 17.7	120.1 ± 14.9	0.5
Recovery (5 min)	90.3 ± 14.5	89.4 ± 15.2	91.2 ± 14	0.63

**Table 4:** The features concerning the patients developing normal and exaggerated blood pressure response to exercise.

	Exaggerated BP response to exercise (+) (*n* = 9)	Exaggerated BP response to exercise (−) (*n* = 51)	*P* value
Group (nebivolol) (*n*, %)	5 (55.5)	25 (49)	0.5
Gender (female) (*n*, %)	7 (77.8)	36 (70.6)	0.5
Age (years)	55.4 ± 8.1	54 ± 11.3	0.7
Smoking (*n*, %)	3 (33.3)	14 (27.4)	0.7
Establishment of targeted resting blood pressure (*n*, %)	3 (33.3)	34 (66.7)	0.065
Systolic blood pressure (mm/Hg)			
Rest	144.5 ± 13.2	135.8 ± 13.3	0.03
Stage 1	165.9 ± 17.3	148.5 ± 22.4	0.01
Stage 2	194.2 ± 22.1	154.8 ± 22.2	<0.001
Stage 3	206.3 ± 31.4	166.3 ± 21.9	<0.001
Recovery (3 min)	178.4 ± 30.5	160.8 ± 25.6	0.04
Recovery (5 min)	166.3 ± 25.4	154.1 ± 28.1	0.17
